# Interaction between science advice and policymaking in time of COVID-19: a French perspective

**DOI:** 10.1093/eurpub/ckac008

**Published:** 2022-01-20

**Authors:** Camille Bruat, Elisabeth Monnet, Jean-Michel Azanowsky, Bernard Faliu, Zeina Mansour, Franck Chauvin

**Affiliations:** 1 Haut Conseil de la Santé Publique (HCSP), Paris, France; 2 UFR Sciences de la Santé, Université de Franche-Comté, Besançon, France; 3 Comité Régional d'Education pour la Santé (CRES) PACA, Marseille, France; 4 Centre Hospitalier Universitaire de Saint-Etienne, Saint-Priest-en-Jarez, France

## Abstract

**Background:**

In the coronavirus disease 2019 (COVID-19) context, many governments relied on scientific consultative bodies to advise their policy, but their contribution remains poorly documented. This article aims to fill this gap by reviewing the role played by the French High Council for Public Health (HCSP) in the French government’s response to COVID-19.

**Methods:**

We studied the time distribution of the COVID-19 guidelines produced by the HCSP until November 2020, computed their delay of production and analyzed the thematic areas they cover. To assess their use by the authorities, we looked for references to these guidelines in the regulatory texts, protocols and press communicates issued by national and local authorities until January 2021.

**Results:**

The HCSP was strongly demanded with 102 guidelines produced following 97 official requests and two self-referrals. Most of them (*N* = 43) concerned protective measures to constrain the infection, while health inequalities and mental health were poorly addressed. Timing was very constraint as half of the guidelines were requested within 4 days. In total, 73% of the guidelines were used by policymakers to implement new obligations or within communication toward the public at national and local levels.

**Conclusions:**

This article informs on the HCSP’s contribution during the crisis and stresses the difficulties it encountered to provide quality recommendations in very short times. It prompts governments to enlarge the competencies of their advisory boards and to consider the multidimensional aspects of health in policy design.

## Introduction

A case of severe acute respiratory syndrome coronavirus 2 (SARS-CoV-2) was officially identified for the first time in the city of Wuhan, China, in December 2019. A few months later, the coronavirus disease 2019 (COVID-19) infection hit the North of Italy and diffused within a few weeks in almost all Europe.^1^ European states were not prepared to respond to the COVID-19 pandemic unlike Asian countries such as China or Taiwan whose warning and response systems had been strengthened after recent outbreaks of emerging diseases.^2^^,^^3^ Still to prevent the diffusion of the virus, governments had to make high-stake decisions in a very constrained time on issues that involve technical considerations, such as testing strategy or vaccination campaign. Yet at the very beginning of the crisis, few data were available on the virus’ proprieties such as its means of transmission, its severity or even the symptoms it triggered in infected people. Then scientific knowledge rapidly evolved, sometimes even shifted, which made it even harder for political leaders to catch the scientific issues at stake.^4^ As a result, many governments relied on scientific bodies to collect and translate information and to interpret its strength and validity.^4^ Taking the form of scientific task forces, panels of experts or scientific committees, these bodies have been central for policymaking during the epidemic in many countries. Some opted to create dedicated COVID-19 task forces to be provided with rapid technical and scientific advice:^5^ Spain for instance set up a Scientific Advisory Committee for COVID-19, bringing together six renowned researchers,^4^ while Belgium established an interdisciplinary task force to advise the country’s Security Council about lockdown lifting.^4^ Newly established bodies appear to comprise a small number of experts so that governments can get information within easy reach^6^ as organizational constraints are reduced.

In the meantime, some governments kept on counting on pre-existing advisory bodies which saw their activity increase during the crisis. The UK government for instance chose to activate a new session of the Scientific Advisory Group for Emergencies (SAGE) to support decision making at the occasion of the Cabinet Office Briefing Room meetings.^7^ The SAGE comprises several sub-groups, each specialized in one specific aspect of the crisis: behaviour, modelling, serology, clinical information, children, hospital, social care or minority ethnic groups. The SAGE includes actors of key public health institutions such as public health England or the National Health Service (NHS) and representatives of relevant government departments.^7^ Belgium for its part relied on a Risk Assessment Group, comprised epidemiologists, scientists and representatives of health authorities, involved in previous crisis managements.^4^

France chose to rely on both pre-established and newly formed entities to inform its COVID-19 policy.

Following the first French COVID-19 cases, the highly publicized autonomous and independent COVID-19 scientific council was set up to update the current state of knowledge on COVID-19 and express opinion on the sanitary measures intended by the government to contain the virus.^8^ Unlike the SAGE, the French Scientific Council operates under government instruction and does not involve government representatives, but external contributors from public health institutions may attend to the meetings as auditors or for hearing. Its workforce is smaller than that of the SAGE with only 13 experts. From March 2020 to January 2021, the Scientific Council delivered 32 guidelines on various topics: sanitary measures to apply throughout the municipal election process, the consequences of virus variants on vaccination or the question of data protection in the context of contact tracing apps for instance. Another newly established advisory body is the Research and Expertise Analysis Committee [Comité Analyse Recherche et Expertise], set up to assess the scientific innovations developed by national and international stakeholders—biotechs, labs or companies—but only guideline has been issued to date.

While benefiting from these ad hoc institutions, France kept on relying on pre-established entities, such as the health agency *S*a*nté* *Publique* *France*, accountable to the Ministry of Health and ensuring the monitoring of the epidemic from an epidemiological point of view. The *Haute Autorité de Santé* (HAS)—an independent public authority—issued recommendations on testing strategy and assessed therapeutics and vaccines efficiency.

As an advisory body, the French High Council for Public Health [Haut Conseil de la Santé Publique (HCSP)] informed the central administration for its COVID-19 policy as well as the President of the Republic, the Prime Minister and other interested ministers at the occasion of the public Health Defence Council meetings, held to coordinate the national crisis policy. The HCSP responds to formal requests coming from ministries and relevant parliament committees on any matter relating to prevention, health security and performance of the health system and may generate internal requests when it deems necessary to do so. Split into four specialized committees on infectious diseases, chronic diseases, the environmental impact on health, and the health system and patient safety^9^ and two permanent task forces for child’s health policy and health inequalities, it brings together nearly 80 independent experts highly qualified in various disciplines of which renowned specialists of the medical field, working as college teachers, doctors in university hospitals or researchers in public labs. While the Scientific Council was rather involved in strategic and steering issues of the COVID-19 crisis, HCSP addressed more operational matters such as therapeutics or hygiene. To meet the demands, it received throughout the crisis, the HCSP created a dedicated ‘COVID-19’ task force on 30 January 2020, consisting of 28 members: a majority of infectious diseases and hygiene medical doctors, 4 public health experts and some environmental engineers and social scientists.

In a situation where the needs of the public authorities in terms of decision support have been important, the contribution of the advisory bodies to the COVID-19 management policy remains poorly documented. To help fill this gap, this article reports the experience of the HCSP, as the authors benefit from direct access to primary data as HCSP’s experts or civil servants. The objectives are (i) to quantify the activity of the HCSP in regard to the evolution of the COVID-19 epidemiology, (ii) to conduct a qualitative analysis of the guidelines produced by the HCSP and (iii) to identify how the public authorities used the guidelines to manage the health crisis.

## Methods

### HCSP’s rules of procedure

Experts’ reports and guidelines are voted by the HCSP’s Board that ensures the principles of expertise are met. For the service of transparency, guidelines are made public 1 month after the commissioning entity receives the guidelines. During this time gap, guidelines remain confidential but the commissioning entity has the possibility to enforce the recommendations and to diffuse them to the public services that might be concerned, including the regional health agencies [Agences Régionales de Santé (ARS)], in charge of the implementation of the national health policy at the regional level. In urgent circumstances, the HCSP’s president alone can endorse the documents’ validation and delay of publication may be reduced conditionally upon the agreement of the commissioning entity. The HCSP’s guidelines are non-binding documents; however, decision-makers cannot modify the guidelines nor interfere with their publication.

### HCSP’s guidelines included in the analysis

We included all COVID-19-related guidelines validated until 12 November 2020 to observe a 2-month delay between the transmission of the last guidelines to the authorities in November 2020 and their potential enforcement (Supplementary table S1). Complementary referrals taking the form of mails or e-mails were excluded.

### Collection of references to the HCSP’s guidelines in official documents

We looked for references to the guidelines in official administrative documents to assess their use by the authorities. The documentary corpus included three types of documents (table 1), covering the regulatory and communication aspects of the authorities' response to the COVID-19 crisis at national and regional levels: the Official Journal of the French Republic (JORF), protocols and press communicates from the central administration and the ARS, and Ministers’ speeches.

**Table 1:** Documentary corpus produced by the French public authorities included in the review

Document	Author	Aim	Source
JORF^a^	French Government	Publishes the new laws and decrees coming into force	Database LégiFrance (www.legifrance.gouv.fr)
Protocols and press communicates from the central political system, Ministers’ speeches	Ministries of Health, Labour, Education, Finance, Culture, French overseas, Armies, Ecological transition, Secretariat of State in charge of Disabled Persons	Protocols provide recommendations for the general population and specific stakeholders (e.g. employers, cultural institutions, health professionals, schools); press communicates and Ministers’ speeches strengthen the diffusion of targeted recommendations	Ministries’ websites (e.g. www.solidarites-sante.gouv.fr for the Ministry of Health)
Protocols and press communicates from the local governance units	The 18 ARSs^b^ that cover each of the France’s regions and overseas areas	The ARSs publish protocols to relay national guidelines. They can be tailored to the local setting. They keep people updated on national and regional regulations and recommendations. Press communicates can be used to emphasize specific messages.	ARS’s websites (e.g. www.iledefrance.ars.sante.fr for the Paris region)

a *Journal Officiel de la République Française* (*Official Journal of the French Republic*).

b *Agences Régionales de Santé* (Regional Health Agencies) in charge of implementing the national health policy at the local level and accountable to the Ministry of Health.

Our search strategy was as follows: we entered the key words ‘Haut Conseil de la Santé Publique’, ‘Haut Conseil de Santé Publique’, ‘Haut Comité de la Santé Publique’ (HCSP’s former name) and ‘HCSP’ in the search engine of official websites (see table 1). The publication time filter was set from 18 February 2020 to 12 January 2021. The research output was manually screened to exclude documents that were not COVID-19 related. References that mentioned HCSP’s guidelines in a not identifiable form were withdrawn. To get an insight into how the HCSP’s guidelines impacted the judiciary branch, we looked for references to the HCSP’s guidelines cited within the *Conseil d’Etat’*s resolutions—the final level of appeal in the French legal system that delivers decisions on administrative litigations happening between a private entity (e.g. citizens, companies) and a public body. To do so, we used the same data collection method, on the *Legifrance* database (‘Administrative jurisprudence’ section).

The queries and data extractions were carried out between 12 January and 20 January 2021.

### Data analysis

We classified the HCSP’s guidelines into seven thematic areas, based on two criteria: (i) the public health field at stake and (ii) the Ministry’s office that initiated the demand. The resulting thematic areas were as follows: (i) individual and collective protective measures (mask-wearing, hand washing and social distancing for the general population and health care facilities); (ii) hygiene and disinfection (cleaning and disinfection of surfaces, sorting of IPE, testing products and masks wastes at home or in health care facilities and adaptation of funeral care); (iii) environmental factors likely to influence the transmissibility of the virus (e.g. ventilation, tobacco, heatwave, swimming water); (iv) blood and organ donation safety; (v) screening and diagnosis including testing strategy; (vi) support for vulnerable populations (disabled and elderly people), health inequalities; (vii) therapeutics and care for COVID-19 patients.

We plotted on a graph the time distribution of the guidelines based on their validation month with regard to the evolution of the disease epidemiology. We chose the weekly COVID-19 hospitalizations as an epidemiologic indicator, as it is independent of the COVID-19 testing rate that has varied along time, available on the French Government website.^10^ We displayed on a box plot the distribution of the guidelines’ expected and observed times of response when the related official request specified a response deadline. The expected time of answer relates to the time between the reception of the request and the response deadline initially set by the commissioning entity; the observed time of response is the difference between the deadline set by the commissioning entity and the effective date of transmission of the document to the commissioning entity. They do not include documents issued at the initiative of the HCSP (synthetic reports and guidelines relating to internal requests). Finally, we computed the number of references according to the guidelines’ thematic area and to the type of official document citing the guidelines.

## Results

**Figure 1:**
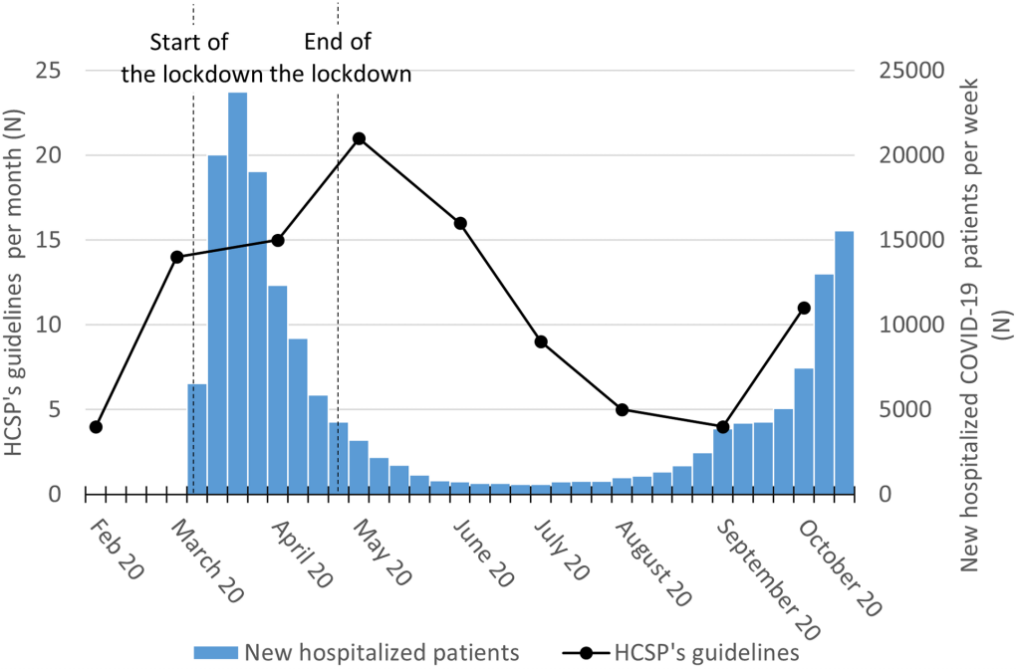
Monthly distribution of COVID-19 guidelines produced by the HCSP from February to November 2020 and number of new hospitalized COVID-19 patients per week (Source: Ministère des Solidarités et de la Santé^10^)

From February to mid-November 2020, the HCSP issued 102 COVID-19-related guidelines following 97 official requests and two self-referrals. The HCSP had been convened twice less over the year 2019 (*N* = 43). Most of the COVID-19 requests came from the Ministry of Health’s biggest division, the ‘Directorate-General of health’ (DGS). One request came from the Secretary of State in charge of children and family attached to the Ministry of Health, and a dozen originated from the Prime minister, the Minister of Health’s office or the Ministry of Education. The guidelines were mainly issued between March and June 2020 (figure 1), a period which includes France’s first lockdown period and its three lifting phases when the expertise was in high demand. We observe a time lag between the peak of hospitalizations and the HCSP’s peak of production during the first wave of the epidemic (March to April 2020), which tends to fade away for the second wave in September 2020 (figure 1). A decrease in production happened during summer when the epidemiological situation improved with decreased new hospitalizations. The guidelines production remained stable following summer when the government relied on the HAS to design the vaccination strategy.

**Figure 2:**
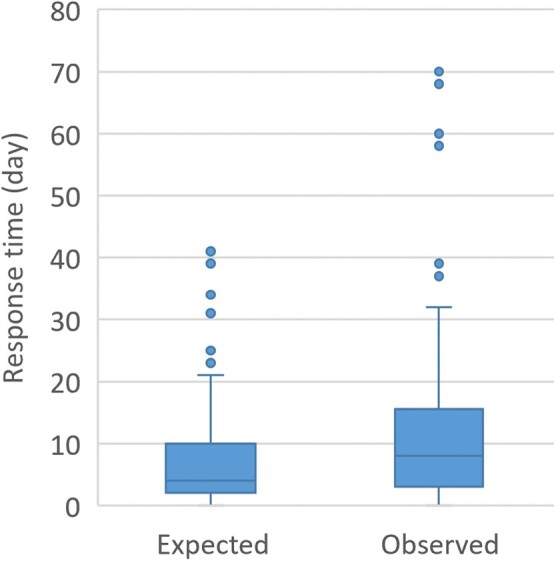
Expected and observed HCSP’s response times to the 88 official requests addressed between February and mid-November 2020 that specified a response deadline

Some 88 guidelines were expected within urgent delay, of which a quarter between 0 and 2 days and a half between 0 and 4 days (median = 4 days; min = 0; max = 41) (figure 2). Yet the HCSP was generally successful in meeting the deadlines (median = 8 days; min = 0; max = 70).

A large part of the guidelines aimed at limiting infections with recommendations on protective measures, hygiene and disinfection as part of primary prevention (table 2, Supplementary table S1). To a lesser extent, some dealt with secondary prevention, with a focus on diagnosis and testing, as well as treatments and care for COVID-19 patients (table 2). Five guidelines addressed health inequalities and designed tailored recommendations for vulnerable populations. Two self-referrals were issued.

**Table 2:** Distribution of the 102 COVID-19 guidelines published by the HCSP from February to mid-November 2020 by thematic area and references to these guidelines found within the official documents published from February 2020 to mid-January 2021

Level of prevention	Thematic area of guidelines, *N*	Total guidelines *N* (%)	Guidelines referenced at least once, *N* (%)	References to the guidelines, *N*			
				Regulatory texts^a^	National protocols and communication^b^	Local protocols and communication^c^	Total
Primary prevention	Protective measures	42 (43)	33 (77)	48	113	32	243
	Cleaning—disinfection	13 (13)	12 (92)	3	12	11	26
	Environment	9 (9)	8 (89)	5	13	33	51
	Donation safety	4 (4)	1 (25)	2	11	9	22
Secondary prevention	Treatment—care	17 (17)	8 (47)	0	1	0	1
	Screening—diagnosis	12 (12)	9 (75)	0	14	14	28
	Support—HI	5 (5)	2 (40)	0	5	0	5
	All areas	102 (100)	73 (71)	58	169	99	326

Note: HI, health inequalities.

a New or updated decrees published in the *Journal Officiel de la République Française*.

b Press communicates issued by the national political system and ministers’ speeches.

c Press communicates issued by the ARS.

The authorities cited almost three-quarters (71%, *N* = 73) of the HCSP’s guidelines in regulatory texts, protocols or communications, for 330 total references (table 2). The HCPS’s guidelines were cited over 50 times in regulatory texts, a majority were on protective measures. A large majority (94%) of the regulatory texts citing the guidelines actually made at least one of the recommendations provided mandatory or enforced provisions going in the same direction. Nearly 270 HCSP’s recommendations were cited in non-coercive protocols, press communicates and/or ministries speeches. Two-thirds of them were diffused by the central administration and one-third by the local health units. The central administration relied on guidelines from each thematic area in a rather homogeneous way, while local health governance units specifically used guidelines on hygiene and environment. We found almost no reference to the five guidelines on health inequalities and vulnerable populations. Similarly, self-referrals were poorly cited (one reference found for two documents).

The 30 guidelines without reference (Supplementary table S1) display higher expected and effective times of response than the overall guidelines (med = 7 days; min = 2; max = 39 and med = 13 days; min = 2; max = 70, respectively). Their distribution by thematic area is proportional to that of the overall guidelines.

We found 65 references to 20 HCSP’s guidelines in the *Conseil d’Etat’*s decisions. Most of the decisions concerned disputes between businesses or citizens and the central administration. They attacked regulatory texts that enforced sanitary measures (wear a mask outside, closure of nightclubs, people limitation indoors) on the ground that they went against the respect of private life, free enterprise or freedom of movement. A majority (74%) of the Conseil d’Etat’s decision rejected citizens' or companies’ requests, on the ground that the sanitary measures were taken based on the HCSP’s guidelines.

## Discussion

Convened twice more during the first COVID-19 months than over the entire year 2019, the HCSP turned out to be an important support for the French authorities throughout the crisis. Its expertise was particularly expected during the first stages of the epidemic, as a reaction to the first peak of hospitalizations. A large number of the resulting recommendations were reflected in regulatory texts at the national level; some were diffused through press communicates, ministries’ speeches and non-coercive protocols from the central administration and the local governance units, but overall, expertise was predominantly used by the central administration, except guidelines relating to the environment thematic area which were mainly diffused by the ARS (this may be explained by the well-developed ARSs’ ‘environmental health’ division that are particularly active in regular time and appear to have maintained their activities during the crisis). This imbalance highlights the centralized crisis management in which expertise is requested and used by the central level that decides on the national policy framework and communication tools further taken up at the local level.

This analysis points out several shortcomings in the collaboration between experts and policymakers.

Firstly, both decision-makers and experts experienced difficulties in apprehending the pandemic through a holistic approach: the HCSP issued many guidelines to protect people’s physical health from potential infections but did not anticipate the pandemic’s side effects on the population’s mental and social health (risks/benefit balance of the lock-down was not accessed).

Few guidelines attempted to mitigate the impacts on health inequalities, which found little or no resonance among decision-makers. This gap may be explained by the nature of the French health system which is heavily care-oriented and often lacks preventive approaches,^11^ what surely contributed to the late resort to the HCSP’s expertise that occurred as a reaction to a high increase in cases. Besides, a narrow range of expertise within the HCSP’s COVID-19 task force has very probably contributed to a medical-centered crisis management. Indeed, few HCSP’s experts benefited from public health training which did not allow to bring up economic or social considerations into the debate. Likewise, the question of the population’s acceptability of the various measures was rarely raised as no mental health professional participated to the council’s meetings. The HCSP is not an isolated case, as the OECD emphasizes many advisory bodies remain predominantly composed of epidemiology, virology, public health and medical experts:^5^ SAGE for instance has raised criticism on its composition, with many clinical practitioners at the detrimental to public health experts or sociologists.^4^ Yet we may wonder whether the decision of implementing strict national lockdowns in many countries would have been taken if the advisory bodies that advised the governments had been composed of specialists in economy, employment or poverty.^6^ Therefore, we believe governments should undertake efforts to bring experts from various disciplines into their national advisory bodies, including social scientists, economists and psychologists. To move forward toward that direction, the HCSP, at the occasion of its 1-year assessment of the COVID-19 crisis, decided to set up a new working group ‘Evaluation, strategy and prospective’: the aim is to develop a public health model that would go beyond virology to anticipate the various components of the pandemic and its progression.^12^ The new working group involves, among others, experts from social sciences and psychology. Such initiatives should be encouraged to prompt governments to produce effective public health policies in which health is considered in its multidisciplinary nature as defined by the World Health Organization.^13^

A second challenge met by the experts is the very constraint time frame in which the guidelines had to be produced. The authorities expected very quick answers which reflect an urgent need as the epidemic was progressing. However, collecting information and debating to provide evidence-based recommendations often requires more time for the experts than the policy-makers have available to make a decision.^5^ Even though deadlines were often met, late returns seem to have impeded the impact of the related guidelines. In addition, the constraint deadlines impacted the collective aspect of the expertise provided. In regular times indeed, the HCSP invites relevant stakeholders for contribution through questionnaires and interviews, but maintaining this participative process was hardly feasible with such deadlines. It is however essential to involve civil society organizations, the private sector and citizens into the decision processes to enhance the relevance and credibility of the measures^14^ and to ensure all determinants behind a public health issue are taken into account. This is particularly the case when a public health decision goes beyond scientific considerations and involves conflicting values (e.g. right to privacy, right to health, freedom of movement),^15^ and when the resulting recommendations are further used to arbitrate conflicts between administration and citizens who vindicate some of those rights. To move forward that direction, it would be relevant to involve representatives of the associative arena (NGO, trade union, worker representatives), as did the French Scientific council with the president of ATD quart monde, an NGO fighting poverty. Representatives of the health sectors should join the debate to represent patients’ rights and health professionals: in France, advisory groups could integrate members of the Health National Conference [*Conférence Nationale de Santé (CNS)]*, an independent public institution in charge of involving representatives of the health system users as well as health stakeholders into the decision process to enhance health democracy.

Finally, several limitations can be found to this study.

Firstly, we limited the review to three official documents that we believe provide fair insight on what has been achieved by the government during the COVID-19 crisis. Yet instructions may have been diffused through internal channels, especially when diffusing from Ministries to the executive health agencies. This was the case for guidelines on blood and organ donation safety that were transmitted to relevant structures (e.g. the French blood institution) through internal networks, what may explain they artificially appeared as unused in our analysis. Complementary analysis such as semi-conductive interviews could provide qualitative information to better identify these channels, but we lacked opportunities to question the central and local administrations due to the workload they have been facing during the crisis. Finally, some of the HCSP’s recommendations may have been used without mentioning the source. As a result, the number of references to the HCSP’s guidelines would have been higher than observed.

## Supplementary data

Supplementary data are available at *EURPUB* online.

## Supplementary Material

ckac008_Supplementary_Data

## Notes

This work should be attributed to the French High Council for Public Health (HCSP).
