# Supplementary material for: Interaction between science advice and policymaking in time of COVID-19: a French perspective
Source: Eur J Public Health. 2022 Jan 20;32(3):468–73. doi: 10.1093/eurpub/ckac008 (PMC9383199; doi:10.1093/eurpub/ckac008)
Supplement: ckac008_Supplementary_Data [file ckac008_supplementary_data.docx]

Supplemental table 1. List of the COVID-19 guidelines produced by the HCSP from February to mid-November 2020 (by validation date)

| Management of a deceased SARS-CoV-2 patient’s body |
| --- |
| Treatment of linen, cleaning of a SARS-CoV-2 confirmed patient’s dwelling or hospital room and protection of personnel |
| Preventive measures to be applied to donors of blood, cells, tissues and organs who have been in at risk area for SARS-CoV-2 transmission |
| Directive for hospital staff returning from at risk areas |
| Management of confirmed SARS-CoV-2 infection cases |
| Patients at risk of severe COVID-19 and prioritisation of diagnostic tests (provisional) |
| Rationalisation of surgical and respirator masks for health professionals for ambulatory care and within health and medico-social establishments during the phase 3 epidemic period |
| Preventive measures to be applied to blood, cells, tissues and organs donors who have been in at risk area for SARS-CoV-2 transmission (complement)* |
| Prevention and management of COVID-19 in patients at risk of severe forms (provisional) |
| Clinical criteria to end isolation of SARS-CoV-2 patients |
| Reducing the risk of SARS-CoV-2 transmission through ventilation and management of COVID-19 patient’s effluent |
| Waste management from healthcare activities in the COVID-19 context |
| Treatments for COVID-19 patients (complement) |
| Management of a deceased suspected of confirmed COVID-19 patient’s body (update) |
| Support for people with disabilities in the context of the Covid-19 epidemic and the extension of lockdown |
| Support for precarious people in the context of the Covid-19 epidemic and the extension of lockdown |
| Prevention and management of patients at risk of severe COVID-19 and prioritisation of diagnostic tests |
| Protection of waste collection workers during the Covid-19 epidemic |
| Appropriateness of specific cleaning or disinfection of the public space |
| Management of suspected or confirmed COVID-19 cases, at home or in care facilities (complement) |
| Waste management of adult incontinence pads used by possible, probable and confirmed COVID-19 patients |
| Residual risk of SARS-CoV-2 transmission by aerosols in health care settings, other indoor environments, and outdoor environments |
| Treatment of linen, cleaning of a SARS-CoV-2 suspected or confirmed patient’s dwelling or hospital room and on the protection of personnel* |
| Child’s health, the Covid-19 epidemic and its impacts |
| Clinical signs for the diagnosis of Covid-19 cases |
| Persons at risk of severe Covid-19 and tailored protective measures |
| Adaptation of protective measures and social distance to be implemented in the general population, outside the health and medico-social fields, to control the spread of SARS-CoV-2 |
| Therapeutic use of convalescent plasma in Covid-19 patients* |
| Access control by temperature measurement for lifting lockdown |
| Public establishments and workplaces cleaning and disinfection before re-opening following the lockdown period |
| Risks associated with 1) the reprocessing of single-use masks, particularly in the health sector, and the possible modalities of their reuse, in the medical and medico-social sectors and for other professional activities outside the health field, and 2) conditions to meet for extending the wearing of masks or other alternatives* |
| Prioritisation of masks for people the most at risk of severe COVID-19 and surgical masks for those at very high risk* |
| Adaptation of protective measures and social distancing in childcare facilities to control the spread of SARS-CoV-2 |
| Bathing water and use of water from the natural environment in the COVID-19 context |
| Lockdown lifting or maintenance for disabled people in the COVID-19 context* |
| Risks of SARS-CoV-2 contamination through textile materials for lifting lockdown |
| Management of the Covid-19 epidemic in the event of population exposure to heat waves |
| Instructions in case of close contact between a person with past symptoms of COVID-19 and a COVID-19 case |
| Link between tobacco and COVID-19 (self-referral) |
| Relevance of RT-PCR pooling for SARS-CoV-2 testing* |
| Use of protective visors or face shields in the COVID-19 context* |
| Physical distancing of passengers on commercial transport aircraft as part of the lockdown lifting and gradual resumption of transport* |
| Remdesivir dose allocation for COVID-19 patients* |
| Use of anti-infectives in COVID-19* |
| Protective measures and physical distancing in commercial catering and drinking establishments (excluding collective catering) |
| Preventive measures for blood, cells, tissues and organs donors in the COVID-19 context (update)* |
| Use of collective misting systems during the COVID-19 lockdown* |
| Protective measures and physical distancing for collective catering (excluding commercial catering) |
| Instructions for professionals working in health, social or medico-social establishments according to their SARS-CoV-2 status |
| Use of hydroxychloroquine in Covid-19 |
| Protective measures and physical distancing in collective accommodation facilities in preparation for reopening in the COVID-19 context (excluding catering and related facilities) |
| Protective measures and physical distancing in cultural spaces in preparation for reopening in the COVID-19 context |
| Provisions likely to be included in the next decree in application to the L3131-15 Article of the Public Health Code on the state of health emergency |
| Protective measures and physical distancing in the context of the resumption of sport activities for the lockdown lifting |
| Relevance of medical and virological check-up before traveling to Corsica in the COVID-19 context* |
| Update of the HCSP’s guidelines of April 24, 2020 on "measures to control the spread of SARS-CoV-2 in schools" in phase 3 of the lockdown lifting |
| Adaptation of barrier and social distancing measures to be implemented in childcare establishments to control the spread of SARS-CoV-2 in phase 3 of the lockdown lifting |
| Relevance of medical and virological check-up before travelling to French overseas in the COVID-19 context |
| Adaptation of protective measures and social distancing to be implemented in school transports in phase 3 of the lockdown lifting |
| Adaptation of protective measures and social distancing to be implemented in schools in phase 3 of the lockdown lifting (synthesis)* |
| Conditions for hosting large-scale events (gatherings of up to 5,000 people) ensuring health safety for the public, the participants and the organization teams, both with regard to Covid-19 and to heat |
| Treatment for Covid-19 patients (update)* |
| Management of the COVID-19 epidemic and social inequalities in child's health, lessons for the future* |
| Adaptation of protective measures and physical distancing in enclosed spaces hosting the public in seated position, in places holding social events (e.g. weddings), in public transport including cruise ships, in phase 3 of the lockdown lifting |
| Adaptation of protective measures and physical distancing in cinemas and enclosed cultural spaces hosting the public in a seated position, in phase 3 of the lockdown lifting* |
| Return to work for people at risk of severe COVID-19 and tailored protective measures |
| Adaptation of protective measures and physical distancing in cabs and car sharing, in phase 3 of the lockdown lifting |
| Draft national lockdown lifting protocol for companies to ensure employees’ safety in phase 3 of the lockdown lifting* |
| Use of ozone, ultraviolet C radiation and airlocks as disinfection processes in the COVID-19 context |
| Remdesivir dose allocation for COVID-19 patients (update)* |
| Support for the resumption of appropriate physical activity for people with chronic diseases and elderly* |
| Relevance of a minimum age for SARS-CoV-2 RT-PCR testing for children before traveling to overseas territories |
| Adaptation of guidelines for schools and universities and for collective reception of minors according to the evolution of the SARS-CoV-2 circulation to prepare school resume in September 2020 |
| Instructions for positive SARS-CoV-2 test in the context of screening or contact-tracing* |
| Possibility of organising gatherings of over 5,000 people and large outdoor dance parties |
| Opportunity for tailored recommendations for populations who may be considered as particularly vulnerable among those at risk of severe COVID-19 |
| Possibility of reopening discotheques in the context of the COVID-19 pandemic* |
| Management of COVID-19 patients (update, report) |
| Scientific knowledge on aerosol transmission of SARS-CoV-2 and related recommendations (update) |
| Access to collective sports changing rooms and practice of outdoor sport activities in the COVID-19 context |
| Relevance of COVID-19 diagnosis from oro-pharyngeal swabs including sputum and relevance of sample pooling* |
| Adaptation of the HCSP’s guidelines in enclosed areas open to the public (particularly in higher education establishments), in the COVID-19 context |
| Adaptation of the HCSP’s guidelines, particularly in public spaces and at large sporting and cultural gatherings, in the COVID-19 context |
| Possible adaptations of the guidelines on masks in enclosed collective places at the workplace |
| Update of the guidelines on masks in childcare facilities for children aged 0-3 years |
| Risk of aerosol transmission of SARS-CoV-2 in healthcare settings |
| Complement to the 9 September 2020 guidelines on strategies to prevent the spread of the SARS-CoV-2 virus in childcare facilities and schools (complement) |
| Preparedness for winter virus outbreaks during the SARS-CoV-2 circulation period |
| Strengthened protocol for restaurants in the COVID-19 context |
| Professional activity of pregnant women in the COVID-19 context |
| COVID-19 screening strategies in health and medico-social institutions* |
| Use of heating appliances in the COVID-19 context |
| COVID-19: use of dexamethasone or other corticosteroid substitutes in hospitalized patients* |
| Protective measures and physical distancing for indoor sport activities in the COVID-19 context* |
| COVID-19 and winter virus in children: preventing and anticipating the saturation of care facilities (self-referral)* |
| Transfer delay of COVID-19 patients towards follow-up care services or retirement homes |
| Preventive measures for cells, tissues and organs donors in the COVID-19 context (update)* |
| Masks in the fight against the spread of SARS-CoV-2 |
| Risk factors for severe COVID-19 (update) |
| Home prescription of dexamethasone (complement)* |
| Management of waste from antigenic tests generated by private health professionals |
| Management of healthcare waste in the COVID-19 context |

*Guidelines with no reference found in the official texts reviewed
